# A massive ovarian mucinous cystadenoma: a case report

**DOI:** 10.1186/1477-7827-8-24

**Published:** 2010-03-11

**Authors:** Remah M Kamel

**Affiliations:** 1 Department of Obstetrics and Gynaecology, Faculty of Medicine, Jazan University, Saudi Arabia

## Abstract

**Objectives:**

To report the occurrence of a rare case of a huge benign ovarian tumour (mucinous cystadenoma) in Jazan city, Saudi Arabia.

**Patients:**

Our reported case was a middle-aged Saudi woman presented with marked abdominal distension and discomfort at the gynaecology clinic of Jazan General Hospital, Jazan city, Saudi Arabia.

**Methods:**

The data were collected by history-taking, clinical examination, laboratory investigations, transabdominal ultrasonographic examination, and by histo-pathological study of the excised surgical specimen.

**Results:**

The case was reported as a rare massive ovarian mucinous cystadenoma.

**Conclusions:**

This case report emphasizes the significance of thorough evaluation of all women presented with vague abdominal pains. Although the condition is extremely rare, it is a potentially dangerous in its massive form if not timely diagnosed and managed properly. With the increasing awareness of such conditions, more and more cases could be detected and reported early.

## Introduction

Ovarian mucinous cystadenoma is a benign tumour that arises from the surface epithelium of the ovary. It is a multilocular cyst with smooth outer and inner surfaces. It tends to be huge in size. Of all ovarian tumours, mucinous tumours comprise 15% [1,2]. About 80% of mucinous tumours are benign, 10% are border-line and 10% are malignant. Although benign ovarian mucinous tumours are rare at the extremities of age, before puberty and after menopause [3], they are common between the third and the fifth decades [4]. The most frequent complications of benign ovarian cysts, *in general*, are torsion, haemorrhage and rupture. As it contains mucinous fluid, its rupture leads to mucinous deposits on the peritoneum (*pseudo-myxoma peritonei*). This report presents a case of a giant ovarian mucinous cystadenoma in a Saudi woman, one of the biggest reported ovarian tumours in the medical literature.

## Case report

A 29-year-old divorced Saudi woman presented with her parents at the gynaecology outpatients' clinic of Jazan General Hospital, Saudi Arabia, with a massive abdominal distension and discomfort. The patient has no living children from her previous marriage that lasted for 3 years. She was divorced one year back. Although the patient and her parents noticed gradual abdominal enlargement since 10 months back, they did not ask for a medical advice as they thought it was a pregnancy. As more than 9 months passed out without commencement of parturition, they consulted their general practitioner (GP) at the primary healthcare unit in their village. The GP suspected a huge abdominal tumour and referred the patient to Jazan General Hospital.

The patient had no previous medical diseases or surgical operations. Her menarche commenced at the age of 13 years with subsequent irregular cycles. She denied the use of any medications.

**Figure 1:**
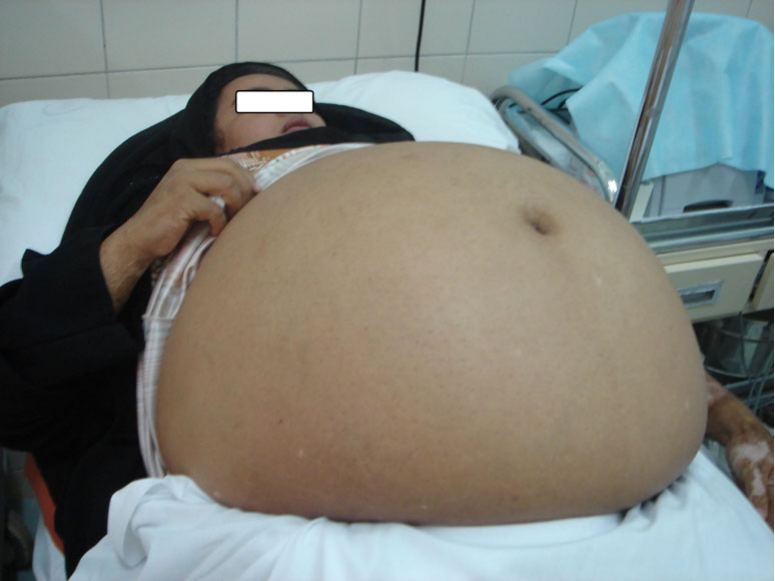
**A giant pelvi-abdominal mass noticed on abdominal examination**.

General examination revealed normal vital signs other than a slight tachypnea (Respiratory rate was 24/minute). Her body weight was 92 kg, her height was 162 cm and her abdominal circumference was 127 cm. Secondary sexual characters were evident. Previous exposure to burn during her childhood left old scars and depigmentation on her upper limbs. On abdominal examination, a huge ill-defined pelvi-abdominal mass was noticed, extended up to xiphisternum, with evident dermal striae. The abdomen was cystic tense on palpation without tenderness or shifting dullness (Figure 1).

**Figure 2:**
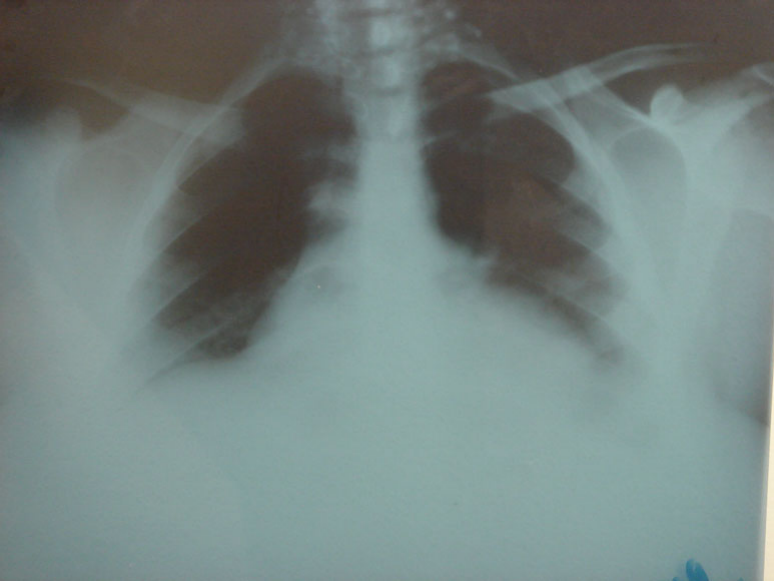
**Plain chest X-ray shows no pleural effusion or metastasis**. Upward displacement of the diaphragm affecting air entry into the lower lobes of lungs (explains patient's tachypnea).

**Figure 3:**
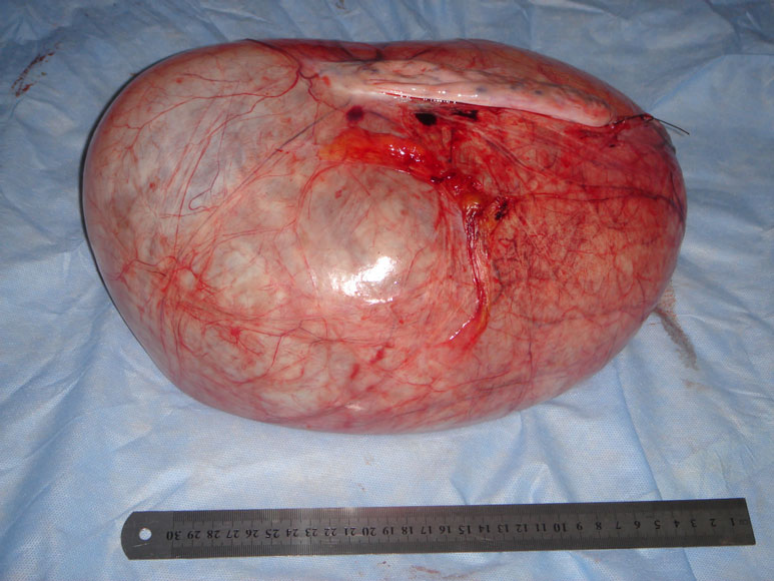
**Gross picture of the intact ovarian tumour shows smooth outer surface without external growths (42 × 28 × 25 cm in diameters and 7,250 kg in weight)**.

**Figure 4:**
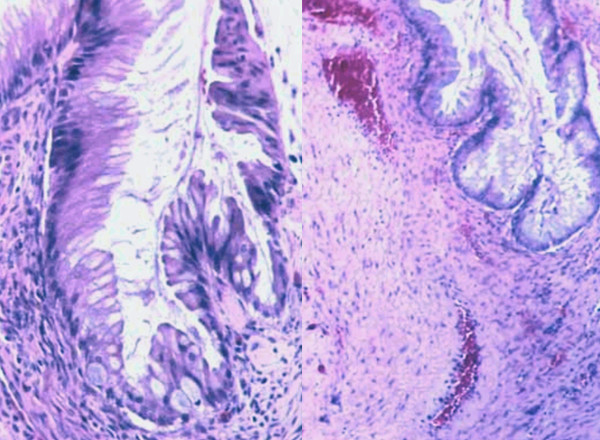
**Microscopic picture of the ovarian tumour shows the lining non-ciliated, *mucin-secreting*, columnar epithelium with goblet cells (Mucinous cystadenoma)**.

Pelvic examination revealed normal sized non-pregnant firm uterus and fullness in the cul-de-sac and both adnexae. Transabdominal ultrasonography verified a massive multi-loculated cyst without solid components or surface papillary projections, extended up to the sub-hepatic area, with minimal free intraperitoneal fluid. The patient was asked to do some laboratory investigations including full blood picture, serum biochemistry, cervical cytology and cancer antigen (Ca-125). A plain chest X-ray on erect position was also done (Figure 2). Our patient was counseled and signed informed consent for surgical exploration. Under general anaesthesia, an initial midline subumbilical incision was done where a huge cystic mass was noticed arising from the left ovary. Later on, the incision was extended up, about 5 cm below xiphisternum, to deliver the cystic mass intact without exposed it to the risk of rupture intraperitoneally. The outer surface of the mass was smooth and intact all-around without external growths or adhesions. The uterus, right adnexa, and appendix were looking healthy. No ascites or enlarged para-aortic lymph nodes were discovered. Left salpingo-oophorectomy was performed as the whole ovary was involved in the mass and the left tube was abnormally dilated and adherent to the mass (Figure 3). The size of the tumour was 42 × 28 × 25 cm with 7,250 kg in weight. Microscopic examination revealed a cyst lined by a single layer of non-ciliated columnar epithelium without stromal invasion, the picture of which is compatible with mucinous cystadenoma (Figure 4). Postoperative recovery was uneventful and the patient was discharged on the 5^th ^postoperative day to be followed-up every 3 months.

## Discussion

Giant ovarian tumours have become rare in current medical practice, as most cases are discovered early during routine check-ups. Detection of ovarian cysts causes considerable worry for women because of fear of malignancy, but fortunately the majority of ovarian cysts are benign.

Mucinous cystadenoma is a benign ovarian tumour. It is reported to occur in middle-aged women. It is rare among adolescents [5] or in association with pregnancy [6]. On gross appearance, mucinous tumours are characterised by cysts of variable sizes without surface invasion. Only 10% of primary mucinous cystadenoma is bilateral [7]. In our case, the tumour was unilateral, affecting the left ovary. The cyst was filled with *sticky *gelatinous fluid rich in glycoprotein. In a previous reported case [6], the tumour weight was 6 kg. In our case, the tumour weighed 7,250 kg.

Histologically, mucinous cystadenoma is lined by tall columnar non-ciliated epithelial cells with apical mucin and basal nuclei. They are classified according to the mucin-producing epithelial cells into three types [4]. The first two, which are always indistinguishable, include endocervical and intestinal epithelia. The third type is the müllerian, which is typically associated with endometriotic cysts [8]. Our case has epithelium of intestinal-like type as many goblet cells were noticed.

Management of ovarian cysts depends on the patient's age, the size of the cyst and its histo-pathological nature. Conservative surgery as ovarian cystectomy and salpingo-oophorectomy is adequate for benign lesions [7]. In our patient, left salpingo-oophorectomy was performed as there was no ovarian tissue left and the tube was unhealthy. After surgery, the patient should be followed-up carefully as some tumours recur [5]. Although the tumour was removed completely and intact with the affected ovary, our patient was given appointments to be reviewed every 3 months for a year.

## Consent

A written informed consent was obtained from her for publication of this case report and its accompanying images. A copy of the written consent is available for review by the Editor-in-Chief of this journal.

## Competing interests

The author declares no competing interests.

## Authors' contributions

This work was done by RMK and there is no contribution of any other authors.

## References

[B1] Vizza E Galati GM Corrado G Atlante M Infante C Sbiroli C Voluminous mucinous cystadenoma of the ovary in a 13-year-old girl J Ped Adoles Gynecol 2005 18 6 419 422 10.1016/j.jpag.2005.09.009 16338609

[B2] Mittal S Gupta N Sharma A Dadhwal V Laparoscopic management of a large recurrent benign mucinous cystadenoma of the ovary Arch Gynecol Obstet 2008 277 4 379 380 10.1007/s00404-007-0556-5 18236062

[B3] Crum CP Lester SC Cotran RS Kumar V, Abbas A, Fausto N, Mitchell R Pathology of female genital system and breast Robbins' Basic pathology 2007 Ch 19 8 Elsevier Company, USA

[B4] Ioffe OB Simsir A Silverberg SG Berek JS, Hacker NF Pathology Practical Gynaecologic Oncology 2000 Lippincott Williams & Wilkins Company 213 214

[B5] Ozgun MT Turkyilmaz C A giant ovarian mucinous cystadenoma in an adolescent: a case report Arch Med Sci 2009 5 2 281 283

[B6] Yenicesu GI Cetin M Arici S A huge ovarian mucinous cystadenoma complicating pregnancy: a case report Cumhuriyet Med J 2009 31 174 177

[B7] Alobaid AS Mucinous cystadenoma of the ovary in a 12-year-old girl Saudi Med J 2008 29 1 126 128 18176687

[B8] Young RH Mills SE, Carter D, Greenson JK, Reuter E The ovary Sternberg's Diagnostic Surgical Pathology 2009 Raven Press, NY 2195

